# Hypothetical *Pneumocystis jirovecii* Transmission from Immunocompetent Carriers to Infant

**DOI:** 10.3201/eid1503.081350

**Published:** 2009-03

**Authors:** Philippe Hauser, Meja Rabodonirina, Gilles Nevez

**Affiliations:** Centre Hospitalier Universitaire Vaudois and University of Lausanne, Lausanne, Switzerland (P. Hauser); Université Claude-Bernard, Hospices Civils de Lyon, Lyon, France (M. Rabodonirina); Université de Brest, Brest, France (G. Nevez)

**Keywords:** Pneumocystis jirovecii, transmission, pneumonia, molecular typing, genotypes, letter

## To the Editor

The recent dispatch article by Rivero et al. reports the transmission of *Pneumocystis jirovecii* from immunocompetent grandparents to their granddaughter (*1*). The authors’ conclusion was based on 2 facts: the grandparents were carriers but neither the parents nor the child’s brother was a carrier, and the *P. jirovecii* genotype observed in the grandparents was identical to that found in the infant. In our opinion, the data provided by the authors do not support the conclusion that transmission has occurred. First, the 2 markers used for typing show a small number of alleles and thus provide low discrimination among isolates (*2*). Consequently, the *P. jirovecii* isolates present in the grandparents and in the infant may have been epidemiologically unrelated. Second, the frequency of occurrence of the different genotypes obtained was not investigated. The presence of the same genotype in the grandparents and in the infant may result from a high frequency of this genotype in the geographic area where the family lived. In fact, the use of a validated typing method and the analysis of unlinked control patients have proven necessary in other studies to demonstrate transmission of *P. jirovecii* (*3*–*7*). We believe that the reported transmission event remains a hypothesis.
