# Supplementary material for: Hypothetical Pneumocystis jirovecii Transmission from Immunocompetent Carriers to Infant
Source: Emerg Infect Dis. 2009 Mar;15(3):506–8. doi: 10.3201/eid1503.081350 (PMC2681130; doi:10.3201/eid1503.081350)
Supplement: Related texts

# Reply: In Response

**Authors:** Laura Rivero, Carmen de la Horra, Marco A. Montes-Cano, Nieves Respaldiza, Vicente Friaza, Rubén Morilla, Sonia Gutiérrez, José M. Varela, Francisco J. Medrano, Enrique J. Calderón

**Affiliations:** Instituto de Biomedicina de Sevilla, Virgen del Rocío University Hospital, Seville, Spain

We appreciate the comments from Hauser and colleagues (*1*) regarding our article that reported a case of *Pneumocystis jirovecii* transmission from colonized grandparents to their infant granddaughter (*2*). We agree with the authors that the 2 markers used for typing, which are described in our article, present a small number of alleles and thus provide low discrimination between isolates. However, these typing methods have been validated and have proven useful for molecular epidemiologic applications in *P. jirovecii* colonization studies (*3*,*4*). Unfortunately, other typing methods that can identify a high number of alleles, such as the sequence analysis of the internal transcribed spacer no. 1 and 2 gene regions, could not be used in our study because a low amplification rate has been observed for these regions when such methods are used to study colonized subjects (*5*). On the other hand, the multitarget single-strand conformation polymorphism method has been used only in patients with *Pneumocystis* pneumonia, and its usefulness for epidemiologic studies in colonized subjects has not been proven (*6*). For our study, we think that genotyping analysis of the mtLSU rRNA gene together with the dihydropteroate synthase (DHPS) gene provided sufficient epidemiologic information because this strategy allows identification of 24 different combinations of genotypes. However, no typing method is able to demonstrate interhuman *P. jirovecii* transmission conclusively because a common environmental source of infection cannot be ruled out in any case. Therefore, as we noted in our article, “We cannot exclude the possibility that the cases described were infected by the same environmental source,” and we only hypothesized that “the infant was infected by *P. jirovecii* through close contact with her grandparents.” However, we continue to think that the airborne transmission of *P. jirovecii* from the grandfather to the grandmother and the infant is the most probable explanation based on genotype data. Also, all persons in close contact with the infant were studied, and only her grandparents were colonized by *P. jirovecii*. Future research is needed to assess the importance of colonized subjects in the *P. jirovecii* transmission to susceptible hosts.

## References

[1] Hauser P, Rabodonirina M, Nevez G. *Pneumocystis jirovecii* transmission from immunocompetent carriers to infant. Emerg Infect Dis. 2009;15:506–7. 10.3201/eid1503.08135019239783PMC2681130

[2] Rivero L, de la Horra C, Montes-Cano MA, Rodríguez-Herrera A, Respaldiza N, Friaza V, *Pneumocystis jirovecii* transmission from immunocompetent carriers to infant. Emerg Infect Dis. 2008;14:1116–8. 10.3201/eid1407.07143118598635PMC2600334

[3] Montes-Cano MA, de la Horra C, Martín-Juan J, Varela JM, Torronteras R, Respaldiza N, *Pneumocystis jirovecii* genotypes in Spanish population. Clin Infect Dis. 2004;39:123–8. 10.1086/42177815206063

[4] Vidal S, de la Horra C, Martín J, Montes-Cano MA, Rodríguez E, Respaldiza N, *Pneumocystis jirovecii* colonization in patients with interstitial lung disease. Clin Microbiol Infect. 2006;12:231–5. 10.1111/j.1469-0691.2005.01337.x16451409

[5] Esteves F, Montes-Cano MA, de la Horra C, Costa MC, Calderon EJ, Antunes F, *Pneumocystis jirovecii* multilocus genotyping profiles in patients from Portugal and Spain. Clin Microbiol Infect. 2008;14:356–62. 10.1111/j.1469-0691.2007.01944.x18261125

[6] Beard CB, Roux P, Nevez G, Hauser PM, Kovacs JA, Unnasch TR, Strain typing methods and molecular epidemiology of *Pneumocystis* pneumonia. Emerg Infect Dis. 2004;10:1729–35.1550425710.3201/eid1010.030981PMC3323257
